# OmixLitMiner 2: Guided Literature Mining for Automated Categorization of Marker Candidates in Omics Studies

**DOI:** 10.1002/pmic.70070

**Published:** 2025-11-02

**Authors:** Antonia Gocke, Bente Siebels, Jelena Navolić, Carla Reinbold, Julia E. Neumann, Stefan Kurtz, Hartmut Schlüter

**Affiliations:** ^1^ Section Mass Spectrometry and Proteomics University Medical Center Hamburg Eppendorf Hamburg Germany; ^2^ Center For Molecular Neurobiology Hamburg (ZMNH) University Medical Center Hamburg Eppendorf Hamburg Germany; ^3^ Department of Cardiology University Heart and Vascular Center Hamburg University Medical Center Hamburg Eppendorf Hamburg Germany; ^4^ University Center of Cardiovascular Science University Heart and Vascular Center Hamburg University Medical Center Hamburg Eppendorf Hamburg Germany; ^5^ Institute of Neuropathology University Medical Center Hamburg‐Eppendorf Hamburg Germany; ^6^ Center For Bioinformatics Hamburg MIN‐Faculty Universität Hamburg Hamburg Germany

**Keywords:** literature mining, OmixLitMiner, proteomics, PubTator3.0, transcriptomics

## Abstract

Omics analyses are crucial for understanding molecular mechanisms in biological research. The vast quantity of detected biomolecules presents a significant challenge in identifying potential biomarkers. Traditional methods rely on labor‐intensive literature mining to extract meaningful insights from long lists of regulated candidates of biomolecules. To address this, we developed OmixLitMiner 2 (OLM2) to improve the efficiency of omics data interpretation, speed up the validation of results and accelerate further evaluation based on the selection of marker candidates for subsequent experiments. The updated tool utilizes UniProt for synonym and protein name retrieval and employs the PubMed database as well as PubTator 3.0 for searching titles or abstracts of available biomedical literature. It allows for advanced keyword‐based searches and provides classification of proteins or genes with respect to their representation in the literature in relation to scientific questions. OLM2 offers improved functionality over the previous version and comes with a user‐friendly Google Colab interface. In comparison to the previous version, OLM2 improves the retrieval of relevant publications and the classification of biomolecules. We use a case study of spatially resolved proteomic data from the mouse brain cortex to demonstrate that the tool significantly reduces the time compared to manual searches and enhances the interpretability of molecular analysis.

**Abbreviations**
GO: gene ontology
GO:BP: gene ontology for biological process
OLM: OmixLitMiner

1

Proteomic, transcriptomic and genomic analyses have become an important part in the investigation of biological processes on the molecular level [1], particularly when comparing different phenotypes or a perturbed biological system with its control. The identification of novel biomarkers or drug targets from omics datasets first requires discovering biomolecules that exhibit significant differences in abundance between distinct phenotypes. However, among the thousands of molecules frequently identified in omics analyses, the number of significantly altered proteins and genes, frequently exceed several hundred, posing a significant challenge for interpretation. Proteins identified in these datasets may already be well known, seldom mentioned or may have never been described with respect to an original scientific question [2]. Biomolecules which fall into the latter two categories—not well known or unknown—could be potential new biomarkers or drug targets and could be the subject of further investigation.

In the past years, several pipelines for automated omics data analysis were developed, mainly focusing on creating protein‐protein interaction networks and performing gene set enrichment analysis [3, 4, 5]. However, these analyses do not replace extensive literature mining for understanding the proteins’ specific function and association in biological processes in the investigated setting. Thus, the development of better literature mining tools could provide substantial benefits to the research community concerning data interpretation and the formulation of scientific questions.

The first crucial step in omics literature mining is the generation of a synonym list, as most genes and proteins are referred to by multiple names. Therefore, prior to undertaking literature searches in databases (e.g., PubMed [6] or Google Scholar [7]), it is essential to compile a comprehensive list of all known gene synonyms. In addition, to focus the search space to the field of interest, context‐dependent keywords should be included in the literature search. In response to these challenges, Steffen et al. (2020) developed OmixLitMiner (OLM) as an automated tool for searching protein lists in PubMed [8]. It employs a scoring method to classify candidates based on existing publications in conjunction with specific keywords into Category 1 (reviews available), Category 2 (publication available), and Category 3 (no publications). Because of modifications to the PubMed application programming interface (API), the initial version of the OmixLitMiner is no longer operational.

Recently, NCBI released PubTator 3.0, a literature resource that employs artificial intelligence to search for genes and proteins in conjunction with diseases and other keywords within the PubMed literature database [9]. The tool provides more detailed results, as it integrates not only the keywords, but also extends the keywords with the same meaning as the original keywords and thus facilitates the mining process for individual candidates. However, PubTator 3.0 handles only one candidate per search, and the biomolecules are not categorized into interpretable classes. Thus, we present the new and improved tool OmixLitMiner 2 (OLM2) to expedite the interpretation of omics data and thereby supporting the identification of potential biomarkers and drug target candidates. While OLM retrieved a list of the gene synonyms, OLM2 also retrieves and adds the protein names to the search queries to fully retrieve the available information, thus enhancing the accuracy and comprehensiveness of the analysis. The input can be a list of identifiers of biomolecules. The literature mining tool searches simultaneously for multiple candidates and significantly reduces the time and effort that is typically required for manual searches of a large number of marker candidates. This is especially aided by the integration of a new user‐friendly Graphical User Interface within Google Colab [10], making the tool more accessible compared to the previously available R package. Furthermore, OmixLitMiner 2 also integrates the sophisticated literature mining capabilities of PubTator 3.0. Table 1 lists the main features of OmixLitMiner and OmixLitMiner 2. In the following, we provide more details on OmixLitMiner 2.

**TABLE 1:** Main features of OmixLitMiner and OmixLitMiner2.

	OmixLitMiner	OmixLitMiner 2
**Implementation language**	R	Python
**Database**	PubMed	PubMed PubTator 3.0
**Searches**	Title + Abstract	Title + Abstract
**Keyword combination**	AND	PubMed: AND PubTator 3.0: and with similar words^a^
**Input**	Accession number or gene name	Accession number, gene name or protein name
**Maximum number of publications retrieved**	1,000	PubMed: 1000 PubTator 3.0: No limit (via link, only 10 retrieved for output table)
**Accessibility**	R package (deprecated)	Google Colab + GitHub
**Categories**	0, 1, 2, 3	1, 2, 3, 4

^a^ Through large language model of PubTator 3.0.

OmixLitMiner 2 is composed of three major components: query building, query retrieval and categorization. It uses two major biomedical data collections: UniProt [11] and the NCBI PubMed database [6]. The software is open source and structured to simplify maintenance in cases of updates, such as new APIs to the input collections. In the Google Colab notebook, users are guided through easy‐to‐fill forms to build the query. First, the list of candidate biomolecules must be provided, along with the format (accession number or gene name). Next, the taxonomy ID of the organism and the keywords, which represent the scientific question being investigated, must be specified. A publication is selected only if it contains all the provided keywords. To build the query, the Uniprot REST API is used to fetch the gene names, synonyms and now newly integrated also the protein names for each protein. The user can choose whether the query should be answered by PubMed or PubTator 3.0. If the user chooses to use PubMed, the gene names, synonyms, and protein names are combined with the keywords provided to form the PubMed query. A user can specify that OmixLitMiner 2 searches for the keywords in only the title or in the title and the abstract. Furthermore, the maximum number of publications to be retrieved and the order according to which the publications are listed can be specified (by date or by relevance). The tool yields an Excel‐file with a list of publications. Due to the limit of 1,048,576 rows in an Excel‐file and not to restrict the number of proteins to be searched, we restricted the number of maximally retrieved publications to 1000. This way, multiple hundreds of proteins can be analyzed by OmixLitMiner 2. If the user chooses to utilize the PubTator 3.0 API, the marker candidates are pre‐processed analogously to PubMed, except for the interaction with the different API. Additionally, no limit is set for the number of publications, as the PubTator 3.0 search API only retrieves the first 10 publications, but links to all search results are provided in the output file.

The query is then supplied to the PubMed search API. OmixLitMiner 2 then parses the list of publications delivered and categorizes the genes/proteins accordingly. If, for a given protein P, the literature retrieval with the query built for P delivers at least one article/publication, P is assigned to either Categories 1 or 2. P is assigned to Category 1 if one of these publications is a review article. If no publication was delivered, but P is a valid identifier for a protein in UniProt, P is assigned to Category 3. In all other cases, P is assigned to Category 4 (Figure 1).

**FIGURE 1:**
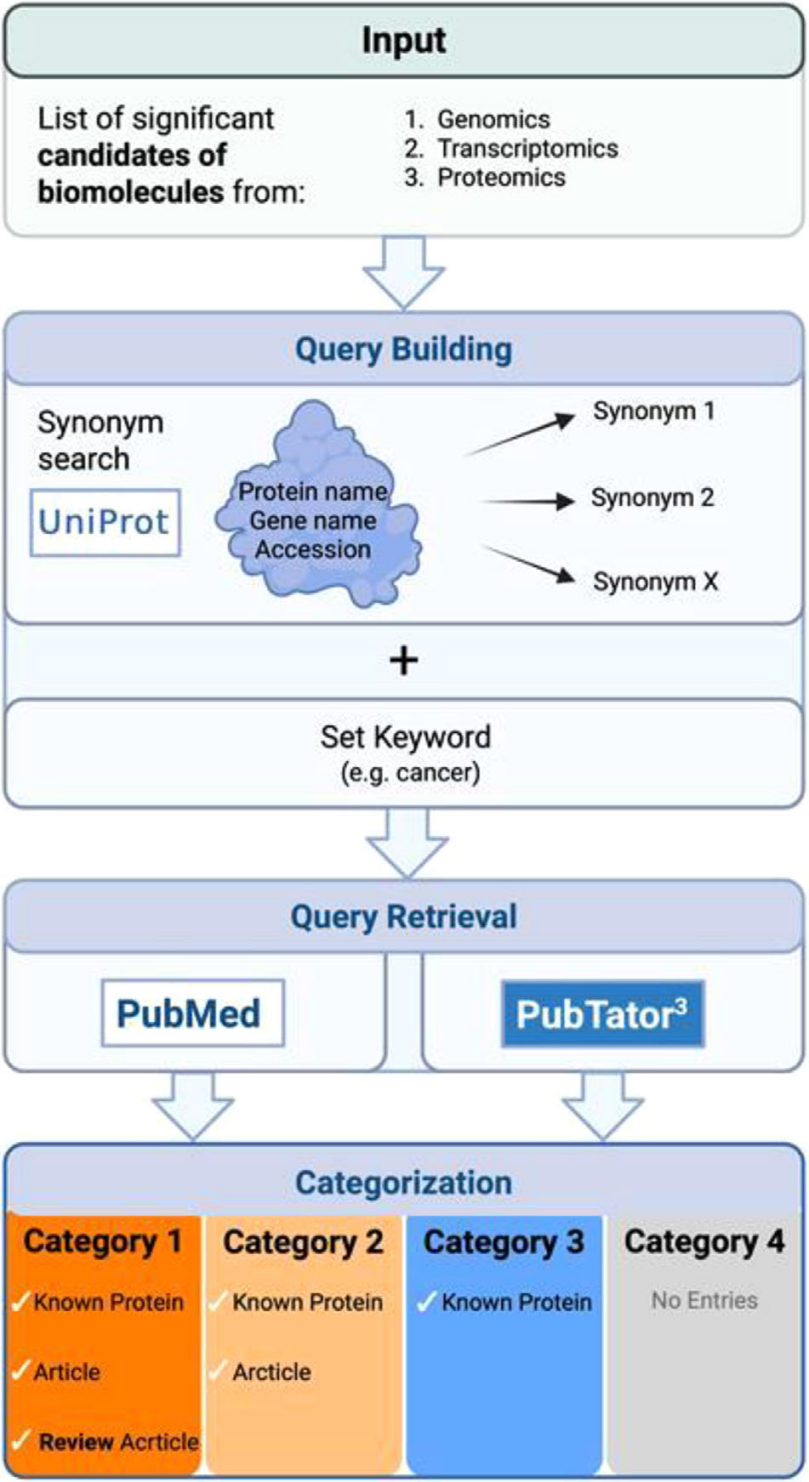
Overview of the literature mining workflow using OmixLitMiner 2. The user provides the input, which is passed to OmixLitMiner 2. In the tool, the list then undergoes query building, query retrieval, and categorization. Created with BioRender.com.

**FIGURE 2:**
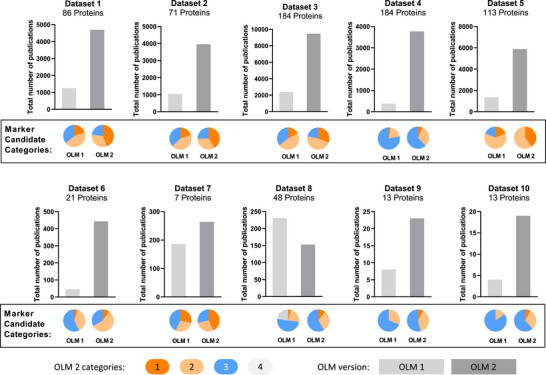
Numbers and categorizations of publications retrieved by OmixLitMiner and OmixLitMiner 2 for datasets 1 to 10. Gene lists, keywords and search results of the OLM were retrieved from the original publication by Steffen et al. Search results of OLM 2 were retrieved based as described by Steffen et al., limiting the search space to all manuscripts published before or on September 13, 2019. For each gene, the number of publications was limited to a maximum of 1000. In the plots, the sum of the number of publications for all gene names in the dataset is shown, with the group distribution indicated in the pie chart below. Category 1: orange, 2: yellow, 3: blue, 4: grey. dataset 1–5: Martinez‐Aguilar et al. [14], dataset 6–7: Hänel et al. [15], dataset 8: Mori et al. [16], dataset 9–10: Tian et al. [17]. Further dataset specifications can be found in Table S1, and detailed results can be found in Tables S2, S3).

While there are literature mining tools that help to retrieve information about specific genes, not many literature mining tools automate the retrieval of publications for multiple genes simultaneously. While GenCLiP 3 [12] also uses biomolecular candidates and certain keywords as an input, instead of classifying the genes, it generates co‐occurrence networks for these genes, indicating possible interactions. Similarly, GIREM [13] also focuses more on reconstructing co‐occurrence networks and integrates the semantic relationship between the co‐occurring genes to generate more accurate networks. Thus, other than OmixLitMiner, there was no other suitable tool against which we could validate our results. However, as the API of PubMed has been updated, we cannot compare OmixLitMiner 2 directly against the OmixLitMiner. The authors of OmixLitMiner published several test datasets, which could be reprocessed with OmxLitMiner 2 by setting a maximum date to the date of retrieval of the original OmixLitMiner publication (September 13, 2019) [8]. The UniProt API integrated in OmixLitMiner 2 only allows for the retrieval of the primary accession number. After the publication of OmixLitMiner in 2020, multiple accession numbers have been deprecated. Thus, we mapped accession numbers to gene names and performed the search based on these. This is our recommended practice for datasets published before 2021. Further specifications of the test datasets can be found in Table S1. The output files with the search results can be found in Table S2.

To evaluate the performance of OLM2 compared to the original OLM, we used the same ten datasets as in the original publication, including proteomics [14, 15, 16] and genomics data [17] and the respective search keywords (e.g., cancer, metastasis, thyroid, migration) depending on the biological context. The results show that OLM2 was able to strongly enhance the sensitivity of the publication retrieval while improving the classification. The latter was shown by considerably larger numbers of proteins assigned to Categories 1 or 2. While the original OmixLitMiner [8] could not search for protein names, the integration of these into OmixLitMiner 2 substantially enhanced the literature retrieval, as in many publications, protein names are used instead of the gene name or accession numbers. For example, in dataset 1, the use of the gene name “DCN” and its synonym “SLRR1” in combination with the keyword “Cancer” only led to the retrieval of two publications. In contrast, the search including the synonyms “DCN”, “SLRR1B”, “Decorin”, “Bone‐proteoglycan‐II”, “PG‐S2”, and “PG40” retrieved 54 publications, including the two publications obtained by a search for only the gene name. None of the 52 additional publications contain the gene name “DCN” or “SLRR1B” in the title, but the protein name “Decorin”. Overall, higher numbers of publications were retrieved when using OmixLitMiner 2, except for dataset 8. For dataset 8, the number of proteins in Category 4 (corresponding to Category 0 in OmixLitMiner) candidates decreased from 11 to 0. That is, for all candidates, synonym searches in UniProt were successful in the sense that no candidates were assigned to Category 0, and no publications containing only the keyword or one of the gene synonyms were retrieved. The high number of publications found by OLM was caused by an erroneous output when the proteins assigned to Category 0 were mapped to 11 publications related to the keyword “metastasis” (Figure 2).

While a PubMed‐based search report publications only if the keyword exactly matches the given term, PubTator 3.0 performs a semantic search, which also delivers publications containing a word that has the same meaning as the given keyword [9]. Using the PubMed and PubTator 3.0 API, literature retrieval was performed for all datasets on the same date (February 28, 2025). In comparison with the PubMed‐based search by OmixLitMiner 2, the PubTator 3.0 based search led to a higher number of retrieved publications with slightly higher numbers of Category 1 proteins, thus highlighting the benefit of the semantic search. We collected the titles and abstracts of the publications retrieved by OmixLitMiner 2 and verified manually that each publication satisfies the search criteria. So, we conclude that the results contain no false positives. For the keyword cancer (in datasets 1, 2, 3, 5, 6, and 7), the newly retrieved papers contained the semantically related word *tumour, tumor*, or *carcinoma*. For all datasets, except dataset 9, the PubTator 3.0‐based search retrieved more publications than the PubMed‐based search (Figure 3). In this dataset, the PubMed‐based search retrieved one publication not found by the PubTator 3.0 based search. However, this publication focuses on a patient suffering from MEN‐1 (multiple endocrine neoplasia 1 syndrome), which is not what we searched for with the protein name “MEN1”. Even though the query to PubMed did not contain the name MEN‐1, the publication was retrieved. In contrast, PubTator 3.0 did not retrieve the wrong publication, again highlighting the benefits of using the semantic search. However, some gene names, like “SCAN” or “DI”, can have different meanings and can be matched to semantically wrong publications (e.g., “Di Bella's method”), even with the PubTator 3.0. Thus, we introduce the concept of *SuperMatch*. A *SuperMatch* is found, when, in addition to the gene name, a protein name can be matched in the title or the abstract (even when searching in title only) in the retrieved publications. The retrieved publication will then show a “Match” in the *SuperMatch* column in the Excel sheet with the results. Otherwise, “noMatch” will be seen instead. With this *SuperMatch*, we hope to reduce the noise of the non‐specific gene names, as protein names are usually more specific than their respective gene names. A low number of *SuperMatches*, but a high number of retrieved publications will most likely be introduced due to a non‐specific gene name, and can be verified manually. An example output can be found in Table S1.7.

**FIGURE 3:**
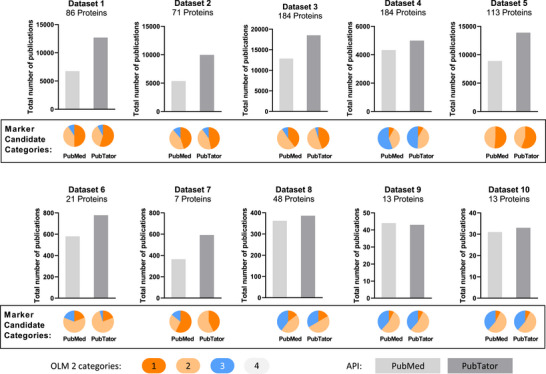
Numbers and categorizations of publications retrieved by OmixLitMiner 2 with the PubMed API and the PubTator3.0 API for datasets 1 to 10. For each gene a maximum of 1000 publications was set. In the plots, the sum of publications for all gene names in the dataset in shown, with the group distribution indicated in the pie chart above. Category 1: orange, 2: yellow, 3: blue, 0: grey. dataset 1–5: Martinez‐Aguilar et al. [14], dataset 6–7: Hänel et al. [15], dataset 8: Mori et al. [16], dataset 9–10: Tian et al. [17]. Further dataset specifications can be found in Table S1, and detailed results can be found in Tables S4, S5).

As a functional validation and an application example, we used data that was published by Navolić et al., 2023 [18]. This data contains the spatially resolved proteome from the scalp to the cerebral cortex of embryonal wild‐type mice into nine layers. Proteins quantified in at least 70 % of the samples were used for t‐testing (limma version 3.54.2), each layer against the other layers (L_n_ vs. L_[_1_,_ 9_] ∖{n}_) to determine higher abundant proteins for each layer. Several significantly higher and lower abundant proteins between the different layers were identified, as visualized in the volcano plot (Figure 4A). These significantly higher abundant proteins (*p* < 0.05; log_2_FC > 1) were selected (SI Appendix Table S1) and categorized using OLM2 combined with keywords covering the main anatomical structures: *skin, bone*, and *cortex*. The PubMed API was used, and the search was restricted to the title and abstract. The proportion of proteins for each category was displayed as a pie plot for the respective layer. The pie plots with the highest number of Category 1 (well‐known) proteins were highlighted by a different colored background (Figure 4A). For each layer, different distributions of Category 1 proteins were retrieved according to the specific keyword (skin, bone, cortex). In line with the known morphology of the mouse head, the highest number of Category 1 proteins was identified for the keyword, which the layer could be mapped to. The first layer could be assigned to skin and bone, then follows one layer assigned to only bone (parietal bone). Layer 3 and layer 4 are correctly not yet assigned to the cortex. Layer 5, which is supposed to be located at the marginal zone and thus has the highest number of Category 1 proteins with the keyword cortex, is wrongly classified as skin. This could be explained by the low number of proteins (two) that were found to be significant for this layer. Lastly, Layers 6–9 could all correctly be assigned to the cortex. While the validation process is used to assign the potential markers to already reviewed literature, a second use case of the OmixLitMiner 2 considers proteins assigned to Category 3 as potentially new marker proteins. This analysis revealed how nuanced distributions of proteins across different tissue layers can be supported by existing literature, and demonstrating the efficacy of OmixLitMiner 2 in facilitating targeted literature mining and functional categorization of proteomic data.

**FIGURE 4:**
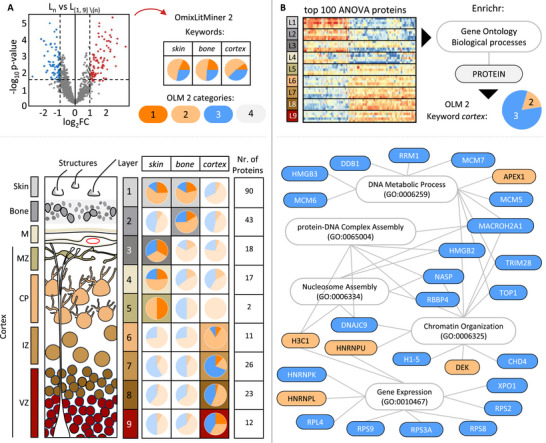
(A) Result of literature Retrieval with OmixLitMiner 2 to validate significantly higher abundant proteins for each layer. Significantly highly abundant proteins upon *t*‐test (*p* < 0.05; log_2_FC > 1) comparing a single layer against the rest of the layers (Ln vs. L [1, 9] ∖{n}) were selected and processed with OLM2 using the keywords: skin, bone, or cortex. The output is displayed as a pie chart labeled with the number of proteins used in the search. Lower panel left: Anatomical structures of the ablated areas from the skin surface to the bone, meninges into the cortex, with the respective pie plot of the result from OmixLitMiner 2 for each Layers 1 to 9. The pie plot with the highest Category 1 publications retrieved is highlighted by a background colored for the respective layer. (B) Categorization of gene ontology associated proteins. Upper panel: Top 100 significant proteins based on ANOVA across Layers 1 to 9 [18]. These proteins were analyzed using gene ontology for biological processes (GO:BP) in Enrichr [19, 20, 21]. Lower panel: Top 5 GO:BP‐terms in white rounded boxes and their related proteins are displayed. Each protein is colored according to the category assigned by OLM 2 when searching with the keyword *cortex*. CP, cortical plate; IZ, intermediate zone; M, meninges; MZ, marginal zone; VZ, ventricular zone.

The top 100 proteins based on ANOVA between all layers were obtained from Navolić et al., 2023 [18] (Table S6). We determined the functional role of these proteins in gene ontology (GO) for biological processes (GO:BP) using Enrichr [19, 20, 21]. The proteins were assigned to different gene sets, and we selected the top 5 GO:BP‐terms, namely DNA Metabolic Process, Chromatin Organization, Gene Expression Nucleosome Assembly and protein‐DNA Complex Assembly (Figure 4B). With OmixLitMiner 2, it is now possible to add a second layer of classification to the proteins. To understand how these are associated with the *cortex*, this was set as a keyword (PubTator 3.0 API and KeywordInTitleOnly = TRUE). The top 5 GO:BP‐terms and related proteins are plotted as a bipartite graph (igraph version 2.0.3), and the proteins are colored according to the category retrieved from OmixLitMiner 2. Proteins that are assigned to Categories 2 or 3 can be considered as less well known in the literature in this context. The proteins from Category 3 are potential candidates for further research questions. Like the proteins MACROH2A1 and DNAJC9, they are linked to multiple GO terms that could be of further interest. Moreover, the integration of such additional information from enrichment analyses has the advantage to reduce the number of potential targets that need to be investigated.

To conclude, OmixLitMiner 2 is an easy‐to‐use tool that rapidly generates a categorized list of publications obtained through automated literature searches involving protein and gene names, as well as keywords related to a specific scientific question. The categorization provides a ranking reflecting how well individual genes or proteins have been studied: Category 1 includes genes and proteins that are well characterized with respect to the scientific question, whereas Category 4 includes proteins that are not yet known or not associated with a gene name. This classification can assist in selecting promising gene or protein candidates for follow‐up experiments. In our validation based on ten different datasets, OLM2 demonstrated increased sensitivity and specificity, as well as an improved user interface compared with the previous version. To further accelerate validation, we implemented the *SuperMatch* approach, which highlights publications in which both gene and protein names were matched, thereby facilitating rapid identification of highly relevant literature.

## Author Contributions

H.S., S.K., and B.S.: conceptualization. A.G. and C.R.: Software OLM2. A.G. and B.S.: software testing. A.G., J.N., and B.S.: validation. A.G. and B.S.: benchmarking. J.N.: case study. A.G. and B.S.: data curation. A.G., B.S., and J.N.: writing – original draft preparation. A.G., B.S., J.N., J.E.N, S.K., and H.S.: writing – review and editing. B.S., J.N., and A.G.: visualization. H.S., S.K., and J.E.N.: supervision. B.S., A.G., and H.S.: project administration.

## Funding

J.E.N. was supported by the Deutsche Forschungsgemeinschaft (DFG, Emmy Noether program).

## Conflicts of Interest

The authors declare no conflicts of interest.

## Supporting information




**Supporting Table 1**: pmic70070‐sup‐0001‐Tables1.xlsx.

**Supporting Table 2**: pmic70070‐sup‐0001‐Tables2.xlsx.

## Data Availability

The OmixLitMiner 2 is available via a google colab notebook (https://colab.research.google.com/drive/1ddpAruLHSX75k1XK9Q1UUZ9sVFdWjp7K?usp=sharing) and via GitHub: https://github.com/AG‐Schlueter/OmixLitMiner2.

## References

[1] C. Manzoni , D. A. Kia , J. Vandrovcova , et al., “Genome, Transcriptome and Proteome: The Rise of Omics Data and Their Integration in Biomedical Sciences,” Briefings in Bioinformatics 19 (2016): 286–302, 10.1093/bib/bbw114. PMC6018996 27881428

[2] M. A. Dar , A. Arafah , K. A. Bhat , et al., “Multiomics Technologies: Role in Disease Biomarker Discoveries and Therapeutics,” Briefings in Functional Genomics 22 (2023): 76–96, 10.1093/bfgp/elac017. 35809340

[3] A. Subramanian , P. Tamayo , V. K. Mootha , et al., “Gene Set Enrichment Analysis: A Knowledge‐Based Approach for Interpreting Genome‐Wide Expression Profiles,” Proceedings National Academy of Science USA 102 (2005): 15545–15550, 10.1073/pnas.0506580102. PMC1239896 16199517

[4] V. K. Mootha , C. M. Lindgren , K.‐F. Eriksson , et al., “PGC‐1α‐Responsive Genes Involved in Oxidative Phosphorylation Are Coordinately Downregulated in Human Diabetes,” Nature Genetics 34 (2003): 267–273, 10.1038/ng1180. 12808457

[5] D. Szklarczyk , R. Kirsch , M. Koutrouli , et al., “The STRING Database in 2023: Protein–Protein Association Networks and Functional Enrichment Analyses for any Sequenced Genome of Interest,” Nucleic Acids Research 51 (2023): D638–D646, 10.1093/nar/gkac1000. 36370105 PMC9825434

[6] PubMed . accessed April 2024, https://pubmed.ncbi.nlm.nih.gov/.

[7] Google Scholar . accessed April 2025, https://scholar.google.de/.

[8] P. Steffen , J. Wu , S. Hariharan , et al., “OmixLitMiner: A Bioinformatics Tool for Prioritizing Biological Leads From ′Omics Data Using Literature Retrieval and Data Mining,” International Journal of Molecular Sciences 21 (2020): 1374, 10.3390/ijms21041374. 32092871 PMC7073124

[9] C.‐H. Wei , A. Allot , P. O.‐T. Lai , et al., “PubTator 3.0: An AI‐Powered Literature Resource for Unlocking Biomedical Knowledge,” Nucleic Acids Research 52 (2024): W540–W546, 10.1093/nar/gkae235. 38572754 PMC11223843

[10] Google Colaboratory—Colab . accessed April 2025, https://colab.research.google.com/.

[11] The UniProt Consortium . UniProt: The Universal Protein Knowledgebase in 2023. Nucleic Acids Research 2023, 51, D523–D531, 10.1093/nar/gkac1052. 36408920 PMC9825514

[12] J.‐H. Wang , L.‐F. Zhao , H.‐F. Wang , et al., “GenCLiP 3: Mining Human Genes′ Functions and Regulatory Networks From PubMed Based on Co‐Occurrences and Natural Language Processing,” Bioinformatics 36 (2020): 1973–1975, 10.1093/bioinformatics/btz807. 31681951

[13] A. Al‐Aamri , K. Taha , Y. Al‐Hammadi , M. Maalouf , and D. Homouz , “Constructing Genetic Networks Using Biomedical Literature and Rare Event Classification,” Scientific Reports 7 (2017): 15784. 29150626 10.1038/s41598-017-16081-2 PMC5694017

[14] J. Martínez‐Aguilar , R. Clifton‐Bligh , and M. P. Molloy , “Proteomics of Thyroid Tumours Provides New Insights Into Their Molecular Composition and Changes Associated With Malignancy,” Scientific Reports 6 (2016): 23660, 10.1038/srep23660. 27025787 PMC4812243

[15] L. Hänel , T. Gosau , H. Maar , et al., “Differential Proteome Analysis of human Neuroblastoma Xenograft Primary Tumors and Matched Spontaneous Distant Metastases,” Scientific Reports 8 (2018): 13986, 10.1038/s41598-018-32236-1. 30228356 PMC6143537

[16] K. Mori , Y. Toiyama , K. Otake , et al., “Successful Identification of a Predictive Biomarker for Lymph Node Metastasis in Colorectal Cancer Using a Proteomic Approach,” Oncotarget 8 (2017): 106935–106947, 10.18632/oncotarget.22149. 29291001 PMC5739786

[17] X. Tian , X. Zhu , T. Yan , et al., “Recurrence‐Associated Gene Signature Optimizes Recurrence‐Free Survival Prediction of Colorectal Cancer,” Molecular Oncology 11 (2017): 1544–1560, 10.1002/1878-0261.12117. 28796930 PMC5664005

[18] J. Navolić , M. Moritz , H. Voß , S. Schlumbohm , Y. Schumann , and J. Hahn , “Direct 3D Sampling of the Embryonic Mouse Head: Layer‐Wise Nanosecond Infrared Laser (NIRL) Ablation From Scalp to Cortex for Spatially Resolved Proteomics,” Analytical Chemistry 95 (2023): 17220–17227, 10.1021/acs.analchem.3c02637. 37956982 PMC10688223

[19] E. Y. Chen , C. M. Tan , Y. Kou , et al., “Enrichr: Interactive and Collaborative HTML5 Gene List Enrichment Analysis Tool,” BMC Bioinformatics [Electronic Resource] 14 (2013): 128, 10.1186/1471-2105-14-128. 23586463 PMC3637064

[20] M. V. Kuleshov , M. R. Jones , A. D. Rouillard , et al., “Enrichr: A Comprehensive Gene Set Enrichment Analysis Web Server 2016 Update,” Nucleic Acids Research 44 (2016): W90–W97, 10.1093/nar/gkw377. 27141961 PMC4987924

[21] Z. Xie , A. Bailey , M. V. Kuleshov , et al., “Gene Set Knowledge Discovery With Enrichr,” Current Protocols 1 (2021): 90, 10.1002/cpz1.90. PMC8152575 33780170

